# Comparison of Early- and Late-Stage Breast and Colorectal Cancer Diagnoses During vs Before the COVID-19 Pandemic

**DOI:** 10.1001/jamanetworkopen.2021.48581

**Published:** 2022-02-15

**Authors:** Jade Zifei Zhou, Shelly Kane, Celia Ramsey, Melody Akhondzadeh, Ananya Banerjee, Rebecca Shatsky, Kathryn Ann Gold

**Affiliations:** 1 Moores Cancer Center at University of California San Diego Health, La Jolla, California

## Abstract

This quality improvement study compares the incidence of early- and late-stage breast and colorectal cancer diagnoses during vs before the COVID-19 pandemic at a California cancer center.

## Introduction

Breast and colorectal cancers are often detected through routine screening of asymptomatic individuals. Effective cancer screening has led to improvements in survival as a result of increased detection of earlier-stage cancer, while decreasing the incidence of late-stage cancer diagnoses.^1,2^ The ongoing COVID-19 pandemic has posed a substantial challenge in cancer care by disrupting cancer screening procedures such as mammograms and colonoscopies.^3^ We sought to compare the incidence of early- and late-stage breast and colorectal cancer diagnoses during vs before the COVID-19 pandemic among patients at our institution.

## Methods

This quality improvement study was approved by the University of California San Diego Aligning and Coordinating Quality Improvement, Research and Evaluation (ACQUIRE) Committee and was deemed exempt from institutional review board approval. Informed consent was waived as per recommendations from the ACQUIRE Committee. The study followed the Standards for Quality Improvement Reporting Excellence (SQUIRE) reporting guideline.

We examined cancer staging for all patients at their first presentation to Moores Cancer Center at University of California San Diego Health for a new diagnosis of malignant neoplasm or a second opinion in 2019 and 2020. To determine the stage at presentation for all patients, the treating clinicians used the American Joint Committee on Cancer staging module (8th edition) in the electronic medical record.^4^ We compared the stage distribution at presentation in 2019 vs 2020 for cancers overall and for colorectal and breast cancers. The Fisher exact test was used to compare the proportions of stage I or stage IV breast cancer, colorectal cancer, and all cancers between 2019 and 2020. Odds ratios (ORs) with 95% CIs and *P* values are reported. The threshold for statistical significance was set at *P* < .05. Statistical analysis was performed with R software (version 3.6.3; R Project for Statistical Computing).

## Results

The study included 55 men (10.5%) and women 467 (89.5%) with a mean (SD) age of 58.1 (13.5) years. Demographic characteristics of the study participants are shown in the Table. Race and ethnicity data were obtained by self-report as part of the standard of care in our electronic medical record. The total number of new patient visits for malignant neoplasm was similar in 2019 vs 2020 (1894 vs 1915). In addition, the overall stage distribution for all patients with cancer was similar, with 605 patients (31.9%) with stage I disease in 2019 vs 556 (29.0%) in 2020 (OR, 1.15 [95% CI, 1.00-1.32]; *P* = .05) and 492 patients (26.0%) with stage IV disease in 2019 vs 506 (26.4%) in 2020 (OR, 0.98 [95% CI, 0.84-1.13]; *P* = .77).

**Table.** Patient Characteristics in 2019 vs 2020

Characteristic	2019, No. (%)		2020, No. (%)	
	Breast cancer (n = 216)	Colorectal cancer (n = 45)	Breast cancer (n = 220)	Colorectal cancer (n = 41)
Biological sex				
Men	1 (0.5)	24 (53.3)	2 (0.9)	28 (68.3)
Women	215 (99.5)	21 (46.7)	218 (99.1)	13 (31.7)
Race^a^				
Asian and Pacific Islander	29 (13.4)	6 (13.3)	23 (10.5)	2 (4.9)
Black	3 (1.4)	1 (2.2)	8 (3.6)	3 (7.3)
White	102 (47.2)	26 (57.8)	120 (54.6)	17 (41.5)
Unreported^b^	82 (38.0)	12 (26.7)	69 (31.4)	19 (46.3)
Ethnicity				
Hispanic	59 (27.3)	14 (31.1)	57 (25.9)	13 (31.7)
Non-Hispanic	157 (72.7)	31 (68.9)	163 (74.1)	28 (68.3)

^a^ Values total 100% each for the race and ethnicity categories, as these data were gathered separately.

^b^ All patients were given the option of whether to specify their race, and this group of patients opted not to report their racial background.

After the start of the COVID-19 pandemic, we saw a numeric but no statistically significant change in the number of patients presenting with stage I colorectal cancer in 2019 vs 2020 (8 [17.8%] vs 6 [14.6%], respectively; OR, 1.26 [95% CI, 0.34-4.88], *P* = .78); the same was true for patients presenting with stage IV disease (3 [6.7%] vs 8 [19.5%], respectively; OR, 0.3 [95% CI, 0.05-1.37], *P* = .11). Among patients with breast cancer, we saw a significantly lower percentage of patients presenting with stage I disease in 2019 vs 2020 after the start of the COVID-19 pandemic (138 [63.9%] vs 116 [51.3%], respectively; OR, 1.67 [95% CI, 1.13-2.47]; *P* = .008) and a significantly higher number of patients presenting with stage IV breast cancer (4 [1.9%] vs 14 [6.2%], respectively; OR, 0.33 [95% CI, 0.09-0.98]; *P* = .04) (Figure). Recent data for January through March 2021 demonstrate a continuing trend of a lower percentage of patients with breast cancer presenting with stage I disease (26 [41.9%]) and an increased percentage of stage IV disease (5 [8.0%]).

**Figure.**
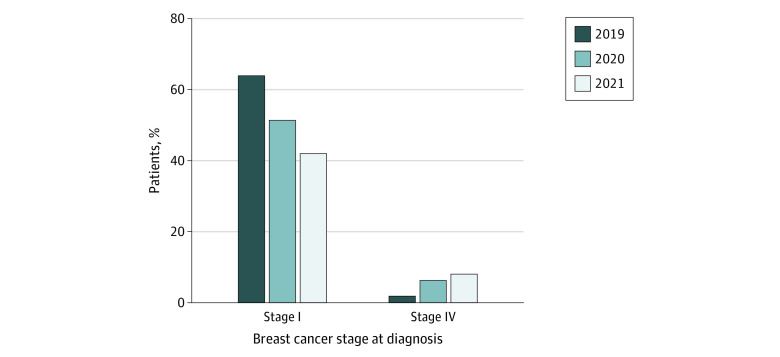
Stage Distribution of Patients With Breast Cancer Presenting for Their Initial Visit in 2019 Compared With 2020 and January to March 2021

## Discussion

The COVID-19 pandemic has profoundly influenced how we deliver cancer care. The results of this quality improvement study suggest that the incidence of late-stage presentation of colorectal and breast cancers at our institution has increased since the start of the pandemic in 2020, corresponding with a decrease in the early-stage presentation of these cancers.

There are several limitations to our study. First, this was a single-center study, and we were unable to assess causality. Second, our numbers of patients with colorectal cancer were relatively small. Finally, our analysis included patients seeking a second opinion, which included individuals with treatment-naive as well as treatment-refractory disease.

Cancer screening is integral to cancer prevention and control, particularly in colorectal and breast cancers. There is increasing concern regarding the effect of the COVID-19 pandemic on cancer mortality, as the evidence suggests that the number of patients presenting at late, incurable stages is increasing. Patients who have delayed preventative care during the pandemic should be encouraged to resume treatment as soon as possible.
